# Putting pharmaceuticals into the wider context of challenges to fish populations in rivers

**DOI:** 10.1098/rstb.2013.0581

**Published:** 2014-11-19

**Authors:** Andrew C. Johnson, John P. Sumpter

**Affiliations:** 1 Centre for Ecology and Hydrology, Wallingford OX10 8BB, UK; 2 Institute for the Environment, Brunel University, Uxbridge UB8 3PH, UK

**Keywords:** fish, chemicals, pollution, habitat, flow

## Abstract

The natural range of fish species in our rivers is related to flow, elevation, temperature, local habitat and connectivity. For over 2000 years, humans have altered to varying degrees the river habitat. In the past 200 years, we added to the environmental disruption by discharging poorly treated sewage, nutrients and industrial waste into our rivers. For many rivers, the low point arrived during the period of 1950s–1970s, when rapid economic development overrode environmental concerns and dissolved oxygen concentrations dropped to zero. In these more enlightened times, gross river pollution is a thing of the past in the Developed World. However, persistent legacy chemical contaminants can be found in fish long after their discharge ceased. Changes in habitat quality and morphology caused and continue to cause the disappearance of fish species. The range of fish stressors has now increased as temperatures rise, and non-native fish introductions bring new diseases. The threat from pharmaceuticals to fish populations remains hypothetical, and no studies have yet linked change in fish populations to exposure.

## 1. Introduction

The exploitation of rivers in the developed Western world is considered to represent a high threat to biodiversity [1], and freshwater fishes are considered among the most threatened group of vertebrates worldwide [2]. Given that fish are vertebrates which share more drug targets with us than other aquatic wildlife, we might expect they would also respond to pharmaceuticals in a similar way [3,4]. This review focuses on the challenges faced by fish in the river environment and tries to put pharmaceuticals in that context. European fish species have preferences for a wide diversity of conditions from cold, fast flowing, highly oxygenated water at one end to warm, slow and low oxygen conditions at the other [5]. Fish also have a wide dietary range and foraging strategies, although most fish larval stages rely on invertebrates [6]. The diversity of species along river networks appears to largely conform to differences in temperature, flow and habitat [7–9]. There are now increasing attempts to use fish to help assess the ecological status of rivers, such as with the ‘European fish index’ (FBI, fish-based index) [10].

## 2. Factors influenced by man that affect fish growth and survival

In our modern landscape, which is so dominated by human activity, changes to the aquatic environment are often due to a combination of both human and natural events. Here, we review factors where human influence has played a role in environmental change, and scientists have connected this to some change in the resident fish populations. What may be disadvantageous to one species may create opportunities for another. It must be acknowledged that in the real world fish are likely to be exposed to multiple coincident environmental stressors that make changes in fish populations very difficult to attribute. The stress on fish can be indirect, such as a reduction in an important food source. Only in acute cases can individual stressors be identified, assuming scientists were also present at the right time and place to witness the change! The FBI process identified 24 potential pressures on fish communities but considered hydrology (flow), morphology (habitat), connectivity (habitat), nutrients (eutrophication) and toxic chemicals/acidification to be the key pressures [10]. Here, we compare nine major factors potentially influencing fish populations against the challenge of pharmaceuticals.

### (a) Flow

The nature of the flow regime is understood to be one of the major components that determine the suitability of a habitat to different fish species [5,7]. Fish which spawn selectively in their natal fast flowing upland streams, such as salmonids, are considered most at risk from man-made changes to flow [7]. The projected lower flows of the future are considered to be unfavourable for salmonids, and other fish whose spawning habitats might get clogged through sedimentation [11,12]. In Spain, declines in brown trout numbers were related to poor recruitment associated with either very low flows or very high flows in the critical month of March [13]. A key factor in the recruitment of fish is survival in the first year and at least for cyprinids this seems to depend on the flow in that year [14,15], with high flow events being particularly hazardous [14,16]. Across France, changes in fish diversity and abundance were most closely correlated to human changes to river flow, including sudden high flows and abstraction, rather than to water quality [17]. Conversely, high flow events leading to flooding may be advantageous for recruitment for some species by reducing the exposure of fry to predation and/or competition in quiet backwaters [18].

### (b) Temperature

Temperature could have a direct stress effect on the physiology of a fish, for example by influencing the sex ratio [19], or indirectly by influencing the abundance of its food source. Some species of fish have temperature-dependent rather than genotypic sex determination, so that a rise in only a few degrees centigrade can dramatically skew the sex ratio of offspring [20]. Salmonids as eggs or juveniles have a narrow temperature tolerance [5]. Consequently, warmer temperatures in the future could become an important stressor for these fish [7]. Warming waters over the past 69 years have been associated with a decline in graylings (a salmonid) in Switzerland [21]. By contrast, warm years are linked to the success of the roach, presumably as they would tend to generate more food for the young fish [14,15].

### (c) Habitat change

It will be appreciated that there are many direct and indirect ways that man's activities could change the quality of a river as a habitat for fish. Early human development was associated with prolific weir fishing and impoundments for mills and forges. This was followed by the straightening and deepening of channels for trade navigation, followed by flood protection levees [22,23]. These interruptions to flow, particularly in the headwaters, were associated with the disappearance of the migratory salmon from the Berlin area of the Elbe from 1787 and the Thames in the 1820s [22]. Other migratory fish, such as the sea lamprey and sturgeon, disappeared in the 1860s [23,24]. The canalization caused habitat changes which were implicated in the subsequent disappearance of barbel and burbot from these rivers [23]. In a review of altered water bodies in Germany (associated with assessment for the European Water Framework Directive), the river bank conditions were considered the most important factor influencing the presence and abundance of fish communities [25]. An investigation into decline of the barbel in the R. Lee in the UK found that man-made river alterations had reduced connectivity which was vital for the development of adult fish [26]. In Spain and North Africa, habitats formerly conducive to eels have suffered drastic habitat changes associated with marsh draining and the construction of dams since the 1980s, making them now unsuitable for these fish [27]. River connectivity, enabling fish immigration and emigration, is also vital for maintaining gene flow and genetic effective population size in salmonid species [28], but in other species, such as roach, this appears to be less important [29].

### (d) Parasites and disease

Outbreaks of disease and parasite infestations have been linked with significant decline in year class success [30,31] and, in exceptional circumstances, a disease can result in mass mortalities [32]. Proliferative kidney disease is considered one of the strongest candidates to explain the decline in brown trout in Swiss rivers [31]: it is caused by *Tetracapsuloides bryosalmonae* which flourishes in warm water temperatures (more than 15°C). Native salmon were reported to have declined by 95% in Norway 7 years after the arrival of *T. bryosalmonae* and the ectoparasite *Gyrodactylus salaris* [33]. In some eel populations, a fish species still in decline, the parasite *Anguillicoloides crassus* is considered a potentially significant danger owing to the associated deterioration of the swimbladder [33–35]. Fish with poor health indicators in polluted rivers in southeast USA were those with parasite infections rather than those with the highest chemical pollutant burdens [36].

### (e) Alien fish introductions

Alien, or non-native, fish have been introduced into European river habitats from the early Middle Ages, beginning with the common carp, followed by such species as the European catfish, pikeperch, rainbow trout in the middle nineteenth century, followed by a new wave of fish species in the 1980s such as sunbleak and topmouth gudgeon [37]. Probably, many of the introduced species did not prosper [37]. There are some suggestions that these intruders have displaced native fish by perhaps being better adapted to existing or evolving habitats [38,39]. But, the presence of alien fish may not be harmful, and indeed, a large recreational industry depends on many of these introduced species [40]. However, the associated introduction of new diseases arriving with the alien fish remains a matter for concern [33,41]. Restocking with ‘native fish’ from fish farms may be detrimental to fish populations, potentially causing loss of genetic diversity, lowered fitness, decreasing return rates and increased susceptibility to disease [42].

### (f) Fishing

Historically, freshwater fish were an important food source, with large nets often stretching across the whole river. But such nets were banned in rivers such as the Thames in 1860 as recreational angling became popular and sea fish could be preserved and sold inland [22]. However, there can be problems with migrating species and recreational anglers. There is an example in Switzerland with grayling and trout populations reducing with the increasing number of fishing licences in some locations [43]. In Spain, the timing of angling intensity was considered to be causing a demographic shift to smaller migrating Atlantic salmon who arrived later in the fishing season. Perhaps not surprisingly, industrial fishing of salmon with gillnets, where it does occur, has been linked to a progressive selection towards smaller-sized fish who could pass through the nets unharmed [44]. However, recreational fishing may sometimes lead to diversifying selection, increasing variability in growth rate and size at age, as shown for pike in Windermere [45].

### (g) Gross organic pollution

The major problem with high organic loading of rivers is the loss of oxygen associated with its consumption by bacteria (the loss of dissolved oxygen downstream of sewage discharge points is frequently called the ‘DO sag’). The early-nineteenth century saw increasing popularity of the flushing toilet, with domestic waste discharge to sewers. As cities grew and in the absence of sewage treatment, this growing waste discharge was very detrimental for rivers. The loss of fish from the lower Thames (UK) was reported in the 1850s and linked to gross sewage pollution [46], with low oxygen remaining an issue in the tidal Thames up to the 1970s [22]. The introduction of some sewage treatment allowed fish to return to the Mersey (UK) in the 1930s–1940s, but by 1950, organic loading was such that the fish had disappeared once more [47]. Even with piecemeal improvements in sewage treatment, dissolved oxygen frequently fell to zero in a 15 km stretch near the tidal limit in the period up to the 1970s [47]. In the Rhine in the 1960s and 1970s, summer dissolved oxygen concentrations dropped to 2 mg l^−1^, an inhibitory level for most fish [48].

### (h) Eutrophication

Between 1921 and 1975, the phosphorus load was estimated to have increased 10-fold in the rivers around Berlin and the associated eutrophication led to the near total loss of submerged macrophytes. This was associated with a decline in phytophilic fish such as pike, carp and tench, but favoured bream [24]. However, more eutrophic conditions from sewage have been associated with increased roach populations in the Baltic region [49]. Roach are described as omnivores, able to eat plant material, invertebrates and molluscs and are unaffected by low light intensities [49]. Thus, eutrophication and associated turbidity could affect fish in several ways, for example by changing the food availability, affecting their ability to find food, predation or even find a mate [50]. Improvements in sewage treatment along the R. Trent from the mid-1970s coincided with declines in roach and dace catches [51]. Over the same period, chub, bream and eel increased. The reduction of phosphate (P) pollution from sewage effluent was considered the key factor in reduced roach growth rates in the R. Wensum in the UK [52]. In contrast to its fellow cyprinid the roach, growth rates of the barbel were positively related to rivers with lower sewage effluent contents (phosphate) [53].

### (i) Metals and toxic chemicals

Metal pollution of rivers began with mining 2000 years ago and then also occurred through direct industrial discharge and atmospheric deposition from combustion processes [54]. Particulate levels of metals were considered to have reached toxic levels in the Rhine in the 1960s [55]. The development of industries added increasing chemical pollutants such as sulfuric acid, metals, cyanides and ammonia to the Mersey basin in the nineteenth century and was such that by 1850 all fish had gone from the river and most of its tributaries [56]. In the Moselle River, its deterioration as an ecosystem began with industrialization in the 1860s. By the 1920s, some important tributaries were described as devoid of life [57].

Fish caught today in the rivers of developed countries typically contain a range of persistent organic pollutants (POPs) that were phased out, or banned, decades ago [36,58]. Eels in Belgium with the highest metals and POPs levels had the lowest condition levels [59]. Given their high fat content and propensity to accumulate organic pollutants, it has been suggested that the dioxin-like polychlorinated biphenyls (PCBs) may be reducing the eel's ability to reproduce by harming the embryos [60]. Studies in the Netherlands and Belgium revealed the decline in eel numbers corresponded with a decline in their fat content. A low fat content may mean it cannot make its trans-ocean migration successfully [61]. Perhaps this decline in fat content is linked to the stress-related demands of POP and metal contamination that reached critical levels in the 1980s [61].

In a study of 117 fish species across 695 sites across Ohio (USA), the potential local effects of the combined mixture of toxic chemicals were compared with other local ecological drivers [62]. The analysis suggested that 50–55% of sites had some chemical toxicity pressure but that on average, over all sampling sites, the relative contribution of chemical mixture effects to local ecological impacts was only 3%. Thus, the assemblages of fish species could be predicted in most cases by factors such as latitude/longitude, slope, habitat and general water chemistry [62]. An alternative way of viewing this result is that if these habitats could be made perfect in every way for the fish, then chemical pollution would then prevent the naturally expected fish assemblage from occurring in half the sites.

### (j) Pharmaceuticals

Fish in the developed world have been exposed to an ever increasing range of pharmaceuticals for at least the past 60 years without dramatic change in their populations being noted. The most consistent and widespread exposure of fish to pharmaceuticals is likely to be from sewage effluent and indeed pharmaceuticals can be found in the bile of wild fish found in proximity to sewage plants [63,64]. It would then follow that if pharmaceuticals harm fish then the worst effects would be seen in rivers with the highest effluent content. With endocrine disruption and reduced breeding potential for individuals, this does appear to be the case [65–67], with the pharmaceutical ethinylestradiol (EE2) likely to be an important contributor [68,69]. We are not aware of any studies showing population effects of pharmaceuticals on wild fish. But are there any messages to be inferred from studies on fish populations in proximity to sewage effluent, or those inadvertently exposed to the highest concentrations of pharmaceuticals during low flows? Roach populations do well in warm summers, which might be considered to be periods of low flows (consequently higher effluent and pharmaceutical contents) [14,15]. Living in rivers with a large treated sewage effluent content does appear to bring its compensations, at least for stickleback and roach, as these fish tend to be bigger and heavier than those where the effluent content (or P content) is much less [52,70]. Thus, to date, there does not seem to be any clear links between the post-1970s regular domestic sewage effluent content of rivers and fish success or failure. Perhaps we have not looked carefully enough, or the issue is complicated by some fish species being more sensitive to certain pharmaceuticals than others?

## 3. Summary

**Figure 1.**
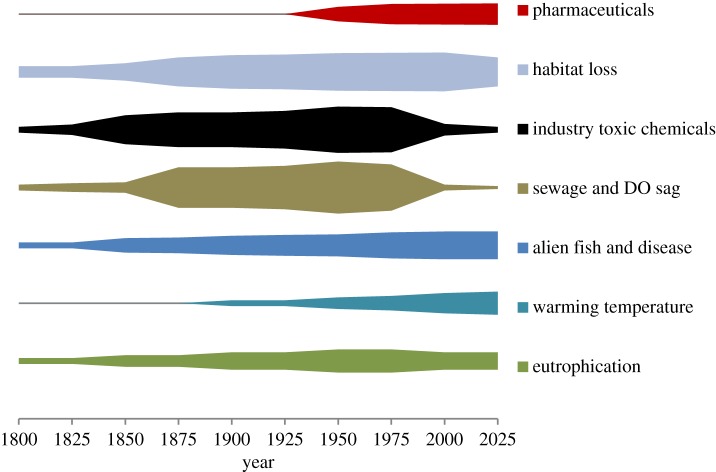
Suggested timeline of stressors faced by fish in urbanized catchments in the Western world and the magnitude of threat they posed. The width of a band at any time point reflects its considered relative impact on fish. The greater the width, the more harmful the impact on fish. (Online version in colour.)

Humans have been changing the environment and inadvertently the resident fish populations in our rivers in a major way for over 1000 years. It is possible to rank these challenges to fish populations (albeit subjectively) through recent history (figure 1). The timing of these deleterious impacts would of course vary between rivers. From what we can understand of our river history, the biggest catastrophes, where fish were wiped out en masse, were related to gross industrial and human waste pollution frequently causing oxygen depletion, and so these factors were ranked highest in the figure. But, we have also learnt that habitat loss can lead to a critical loss of species. The ‘perfect storm’ of poorly treated sewage, toxic industrial chemicals and habitat loss, which peaked for many rivers between 1950 and 1975, has now passed, although lethal accidental spills can still occur [71]. While the biggest threats to fish survival may now be history, the range of stressors has increased, with a new one, pharmaceuticals, having recently appeared. We have evidence that in combination, or even alone, gross sewage or toxic chemicals pollution have on occasions eliminated all fish from a river. Evidence also exists for changes in habitat, flow and eutrophication as capable of causing changes in species diversity in rivers. Warming temperatures, introduced diseases and some toxic chemicals may be harming, but not necessarily currently eliminating, fish species. As yet the risk to fish populations from pharmaceuticals, acting both independently and in combination with each other and with different stressors, remains hypothetical. The literature on pharmaceuticals and fish is dominated by laboratory and caged fish studies, where a range of effects and potentially harmful endpoints have been reported. But, until harmful effects on fish populations in the wild are identified, pharmaceuticals cannot be ranked as one of the most dangerous challenges to fish. The apparent absence of evidence for fish population damage from pharmaceuticals should not, however, lead us to complacency for the following reasons:

— the evidence may be there, but we have not collected it in a systematic way;
— future damage to fish populations may occur if we exceed a threshold level owing to reduced flows or increased human/veterinary consumption. For EE2, this would need to be a concentration rise of only 10-fold;
— mixtures of similarly acting pharmaceuticals may be already, or close to, having effects where a single compound may be ignored; and
— more potent pharmaceuticals may enter the market and ultimately the aquatic environment.
